# Impact of non-alcoholic fatty liver disease and liver fibrosis on outcomes of acute ischemic stroke: A systematic review and meta-analysis

**DOI:** 10.12669/pjms.41.2.10729

**Published:** 2025-02

**Authors:** Liting Yang, Jian Han, Chenghui Qin, Feifeng Song

**Affiliations:** 1 Liting Yang Department of Neurology, Affiliated Hospital of Shaoxing University, Shaoxing, Zhejiang Province 312000, P.R. China; 2 Jian Han Department of Neurology, Affiliated Hospital of Shaoxing University, Shaoxing, Zhejiang Province 312000, P.R. China; 3 Chenghui Qin Department of Neurology, Affiliated Hospital of Shaoxing University, Shaoxing, Zhejiang Province 312000, P.R. China; 4 Feifeng Song Department of Neurology, General Hospital of Shaoxing Second Hospital, Shaoxing, Zhejiang Province 312000, P.R. China

**Keywords:** Liver steatosis, Mortality, Steatohepatitis, Stroke

## Abstract

**Objective:**

We reviewed the evidence on the impact of non-alcoholic fatty liver disease (NAFLD) and liver fibrosis on mortality, functional dependence, and recurrence after acute ischemic stroke (AIS).

**Methods:**

This PROSPERO registered review searched PubMed, Embase, CENTRAL, and Web of Science databases from inception of databases to 30^th^ July 2023 for studies comparing outcomes of AIS based on the presence of NAFLD and liver fibrosis. Adjusted data on mortality, functional dependence and risk of recurrent AIS was pooled to obtain odds ratio (OR) with 95% confidence intervals (CI) in a random-effects model

**Results:**

Ten studies were included. Descriptive analysis showed conflicting effects of NAFLD on AIS outcomes with some studies showing better functional outcomes with the presence of NAFLD. Meta-analysis showed that the presence of liver fibrosis was associated with a significantly increased risk of mortality (OR: 2.22 95% CI: 1.02–4.86 I^2^=92%) and functional dependence (OR: 1.89 95% CI: 1.27–2.82 I^2^=53%) as compared to no fibrosis. Meta-analysis found that liver fibrosis did not increase the risk of recurrent AIS (OR: 1.32 95% CI: 0.74–2.37 I^2^=74%).

**Conclusion:**

Scant evidence exists for the effect of NAFLD and liver fibrosis on AIS outcomes. A paradoxical effect of NAFLD on functional outcomes has been noted which needs confirmation by future studies. Liver fibrosis was found to increase the risk of mortality and functional dependence in AIS.

## INTRODUCTION

Non-alcoholic fatty liver disease (NAFLD) is characterized by excessive accumulation of liver fat (>5%) which is not attributable to increased alcohol consumption, steatogenic medications, viral infections, or other coexisting liver diseases.^1^ The disease is one of the leading causes of cirrhosis around the world.^2^ Recently, the definition of the disease has changed from NAFLD to metabolic dysfunction-associated fatty liver disease (MAFLD) which includes hepatic steatosis with either metabolic abnormalities, diabetes, or overweight/obesity.^3^

NAFLD has two primary subtypes namely non-alcoholic fatty liver and non-alcoholic steatohepatitis. The latter is an advanced stage defined histologically with the presence of hepatocyte ballooning and lobular inflammation in addition to steatosis.^4^ The next stage of the spectrum is liver fibrosis which occurs with subsequent collagen deposition and vascular remodeling. The presence of liver fibrosis has been shown to exponentially increases the risk of liver-related as well as all-cause mortality in such patients.^5^ A recent large cohort study has also demonstrated that patients with biopsy-proven NAFLD have an increased risk of adverse cardiovascular events like acute ischemic stroke (AIS), heart failure, and ischemic heart disease along with heightened risk of cardiovascular mortality.^6^ Another study demonstrates that liver fibrosis scores are associated with a significantly increased risk of stroke.^7^

Given the high prevalence of NAFLD in the world population^8^ and the heightened risk of AIS in such patients, it would be interesting to understand the impact of NAFLD and liver fibrosis on outcomes of AIS. To date, no systematic review has been conducted to examine this clinical question. Hence, the present study was undertaken to analyze the effect of NALFD and liver fibrosis on outcomes of AIS.

## METHODS

Protocol registration was done on PROSPERO and the review was allotted the number CRD42023444649. For inclusion in the review studies were to be:

### Inclusion Criteria:

- Conducted on the population of AIS with or without TIA.
- Comparing mortality, functional dependence, or recurrence between patients with and without NAFLD or liver fibrosis.
- Reporting the outcomes as adjusted effect size or as dichotomous data.

### Exclusion Criteria:

- Studies not dividing patients into two groups based on the presence and absence of NAFLD or liver fibrosis based on a clear definition were excluded.
- Studies not reporting on any of the above-mentioned outcomes, duplicate studies, and non-comparative studies were also not eligible.

### Search source and strategy:

English language studies for the review were identified by a literature search conducted on PubMed, Embase, CENTRAL, and Web of Science. Two reviewers (LY & JH) searched articles published from inception of databases to 30^th^ July 2023. Also, the bibliography of the final included studies was hand-searched for any missed articles. Keywords used were “NAFLD”, “MAFLD”, “non-alcoholic fatty liver disease”, “liver steatosis”, “steatohepatitis”, “liver fibrosis”, AND “stroke”. The search string was: (Non-alcoholic fatty liver disease) OR (NAFLD)) OR (liver steatosis)) OR (steatohepatitis)) OR (liver fibrosis)) OR (MAFLD)) AND (stroke). This was also replicated across the different databases. Two investigators (LY & JH) separately examined the titles and abstracts of searched studies after electronic deduplication. Studies relevant to the review were identified while non-relevant articles were excluded. Selected studies underwent full-text analysis against the inclusion criteria. All discords between reviewers were solved by discussion.

### Extracted data and Risk of bias analysis:

Two reviewers (JH & CQ) independently extracted relevant information from the studies which included: the name of the first author, publication year, location of the study, included patients, assessment of NAFLD or liver fibrosis, cut-off values, sample size, demographic details, diabetes mellitus, hypertension, initial National Institute of Health Stroke Scale, outcome data, and follow-up. Outcomes of interest were mortality, functional dependence, and recurrence of AIS. Functional dependence was defined as a modified ranking scale (mRS) of 3-6. Two reviewers (JH & CQ) assessed the methodological quality of the observational studies by the Newcastle Ottawa Scale (NOS).^9^ Points were awarded for representativeness of the study cohort, comparability of groups, and measurement of outcomes.

### Statistical analysis:

PRISMA reporting guidelines were followed.^10^ The meta-analysis was done on “Review Manager” (RevMan, version 5.3). Sufficient studies were available only for the meta-analysis based on the presence and absence of liver fibrosis. Adjusted data was entered into the software to derive pooled odds ratio (OR) with 95% confidence intervals (CI) in a random-effects model. Results were presented as forest plots. Outliners were assessed using a sensitivity analysis involving the removal of one study at a time. Due to too few studies, publication bias was not checked. The chi-square-based Q statistics and I^2^ statistic was used for inter-study heterogeneity. A p-value of <0.10 for Q statistic and I^2^ >50% meant substantial heterogeneity. Due to differences in data presentation amongst studies on NAFLD, only a descriptive analysis was conducted.

## RESULTS

**Fig.1:**
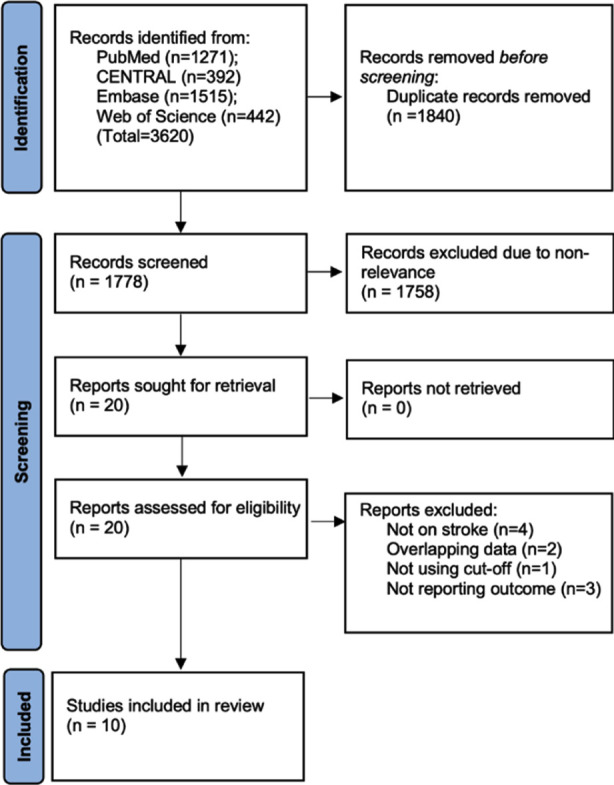
Study flowchart.

The entire literature search revealed 3620 articles (Fig.1). Based on the eligibility criteria, 10 were selected for inclusion.^11^-^20^ Details of four studies assessing AIS outcomes based on the presence of NAFLD. There were three retrospective and one prospective study (Table-I). NAFLD was diagnosed based on aminotransferase levels, fatty liver index (FLI), and transient elastography (TE). The proportion of diabetics was higher in the NAFLD group vs the control group in three of the four studies. Follow-up ranged from discharge to one year the NOS scores varied from 6-8.

**Table-I:** Details of included studies for NAFLD.

Study	Location	Study type	Included patients	NAFLD marker	Cut-off	Groups	Sample size	Age	Male gender	DM (%)	HT (%)	Median NIHSS	Outcomes	Follow-up	NOS score
Chen 2023^13^	China	R	All AIS or TIA patients admitted within 7 days of onset	FLI	FLI ≥35 for males and FLI ≥ 20 for females	NAFLD No NAFLD	3610 2039	61.4 65.2	56.4 78.2	28.6 19	68.8 56.2	NR	Mortality, functional dependence	1 year	8
Baik 2019[14]	Korea	R	First AIS or TIA	CAP on TE	>222 dB/m	NAFLD No NAFLD	206 115	66 70	60.7 57.4	35 28.7	76.7 64.3	2[0-4] 3[1-6]	Functional dependence	3 months	7
Abdeldyem 2017[12]	Egypt	P	All AIS	ALT and/or AST	Above the upper limit of normal in the absence of other causes	NAFLD No NAFLD	85 115	65.4 66.5	52.9 56.5	61.9 30.4	80 81	NR	Functional dependence	Till discharge	6
Tziomalos 2013[11]	Greece	P	All AIS	ALT and/or AST	Above the upper limit of normal in the absence of other causes	NAFLD No NAFLD	32 383	77.4 78.8	40 37.1	24 31.8	80 82.5	6.3± 6.4* 8.8± 9.6	Mortality, functional dependence	Till discharge	6

AIS, acute ischemic stroke; TIA, transient ischemic attack; CAP, controlled attenuation parameter; TE, transient elastography; FLI, fatty liver index; DM, diabetes mellitus; HT, hypertension; NIHSS, National Institute of Health Stroke Scale; ALT, alanine aminotransferase; AST, aspartate aminotransferase; P, prospective; R, retrospective; NAFLD, non-alcoholic fatty liver disease; NOS, Newcastle Ottawa Scale

* Mean value.

Due to the heterogeneous data presentation, a meta-analysis was not carried out for the impact of NAFLD on the outcomes of AIS. Chen et al.^13^ reported data on mortality and functional dependence based on the presence of NAFLD. The authors noted that in comparison to no NAFLD, the presence of NAFLD was associated with reduced risk of functional dependence (adjusted, OR: 0.72 95% CI: 0.61-0.86) and mortality (adjusted, OR: 0.64 95% CI: 0.48-0.85) at one year of follow-up. Baik et al^14^ also found a similar paradoxical effect of NAFLD on functional outcomes after AIS. Their study found that NAFLD was associated with 2.5 times better odds of favorable functional outcome (mRS= 0-2) (adjusted, OR: 2.45 95% CI: 1.08–5.67). Contrastingly, Abdeldyem et al^12^ have shown that the mean mRS score at discharge was significantly higher in NALFD patients as compared to controls (3.6± 2.3 vs 1.8± 2.4). Tziomalos et al.^11^ in their study have shown that mRS scores at discharge (1.9± 2.2 vs 2.6± 2.2) and in-hospital mortality did not differ between NAFLD vs control groups.

Details of the six studies on liver fibrosis are shown in Table-II. Except for one study, all were prospective. Two studies included patients treated only with intravenous thrombolysis. All studies used the fibrosis score for assessing liver fibrosis. Two studies used a cut-off of 3.25 while the remaining studies used a cut-off of 2.67. The median National Institute of Health Stroke Scale (NIHSS) score was also higher in the fibrosis group as compared to the control group. Follow-up ranged from three to 16.3 months the NOS scores varied from 7-9.

**Table-II:** Details of included studies for liver fibrosis.

Study	Location	Study type	Included patients	Liver fibrosis marker	Cut-off	Groups	Sample size	Age	Male gender	DM (%)	HT (%)	Median NIHSS	Outcomes	Follow-up	NOS score
Toh 2023[19]	Singapore	R	AIS treated with IV tPA	FIB-4	2.67	Fibrosis No fibrosis	161 342	81 56	50.3 65.2	75.2 56.7	5 20.5	21[16-24] 12[7-19]	Mortality, functional dependence	3 months	8
Norata 2023[17]	Italy	P	AIS treated with IV tPA	FIB-4	2.67	Fibrosis No fibrosis	32 232	NR	NR	NR	NR	NR	Functional dependence	3 months	7
Canillas 2022[20]	Spain	P	AIS patients aged 40-79 years	FIB-4	2.67	Fibrosis No fibrosis	NR	NR	NR	NR	NR	NR	Functional dependence	3 months	8
Kim 2022[16]	Korea	P	All AIS patients admitted within 7 days of onset	FIB-4	3.25	Fibrosis	670	79	51.8	24.6	67.5	10[4-16] 7[2-15]	Mortality, recurrence	16.3 months	9
						No fibrosis	2227	73	51.8	32.3	69.7				
Fandler-Höfler 2021[15]	Austria	P	AIS with large vessel occlusion	FIB-4	2.67	Fibrosis	104	77	50	16.3	77.9	16[12-19] 14[11-17]	Mortality, functional dependence	3 months	8
						No fibrosis	356	66.7	50.8	16.9	67.7				
Baik 2020[18]	Korea	P	First stroke or TIA	FIB-4	3.25	Fibrosis	231	76.3	53.2	30.7	76.2	3[1-10] 2[1-4]	Mortality, recurrence	1.2 years	9
						No fibrosis	1129	58.5	58.4	32.7	67.6				

AIS, acute ischemic stroke; TIA, transient ischemic attack; IV, intra venous; tPA, tissue plasminogen activator; FIB, fibrosis score; NR, not reported; DM, diabetes mellitus; HT, hypertension; NIHSS, National Institute of Health Stroke Scale; P, prospective; R, retrospective; NOS, Newcastle Ottawa Scale

**Fig.2:**
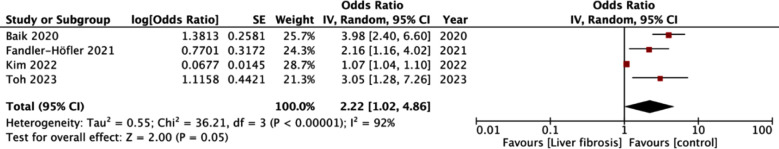
Meta-analysis of risk of mortality after AIS based on the presence of liver fibrosis.

**Fig.3:**
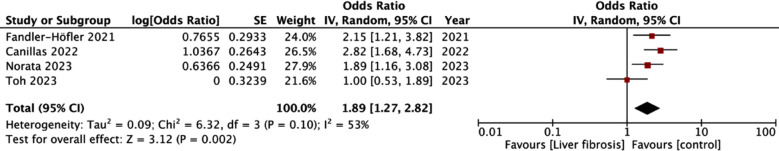
Meta-analysis of risk of functional dependence after AIS based on the presence of liver fibrosis.

**Fig.4:**
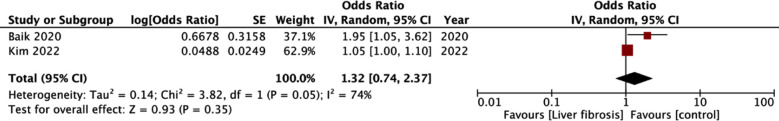
Meta-analysis of risk of recurrence after AIS based on the presence of liver fibrosis.

Meta-analysis showed that the presence of liver fibrosis was associated with an increased risk of mortality as compared to no fibrosis (OR: 2.22 95% CI: 1.02–4.86 I^2^=92%) (Fig.2). The OR of 2.22 indicates that patients with liver fibrosis had a 2.2 times higher risk of mortality as compared to those without liver fibrosis. Pooled analysis showed an increased risk of functional dependence with the presence of liver fibrosis (OR: 1.89 95% CI: 1.27–2.82 I^2^=53%) (Fig.3). The OR of 1.89 indicates a 1.89 times increased risk of functional dependence in patient with liver fibrosis. Meta-analysis found that liver fibrosis did not increase the risk of recurrent AIS (OR: 1.32 95% CI: 0.74–2.37 I^2^=74%) (Fig.4). The results indicate a 1.32 times higher risk of recurrent AIS in patients with liver fibrosis.

## DISCUSSION

The high prevalence of NAFLD and metabolic diseases combined has led to the definition of MAFLD wherein hepatic steatosis is associated with either obesity, Type-2 diabetes, or other metabolic dysfunctions.^3^ Additionally, evidence also suggests that NAFLD may be associated with a higher risk of cardiovascular events independent of metabolic comorbidities.^6^,^7^,^21^,^22^ A pooled analysis has demonstrated 2.3 times increased risk of cerebrovascular accidents in patients with NAFLD.^22^ However, recent evidence has provided some contradictory results. Alexander et al.^23^ have shown that NAFLD was not associated with an increased risk of myocardial infarction or stroke after adjustment of cardiovascular risk factors. Wu et al.^24^ have also found no possible causal effect of NAFLD on stroke. Such inconsistency is also noted regarding the effect of NAFLD on stroke outcomes.

Out of the four studies available for review, two reported data on mortality while all reported data on functional outcomes. The adjusted and long-term data of Chen et al.^13^ found no difference in mortality with or without the presence of NAFLD while Tziomalos et al.^11^ noted no difference in in-hospital mortality between the two groups (unadjusted data). Interestingly, a stark difference was noted amongst studies for functional outcomes. Two studies^13^,^14^ with adjusted data showed a paradoxical effect of NAFLD on functional outcomes with better mRS scores in patients with NAFLD. On the other hand, two studies^11^,^12^ with unadjusted data found either no difference in outcomes or worse mRS scores with NAFLD.

There could be several possible reasons for such inconsistencies. First, the latter studies^11^,^12^ were of small sample size. Secondly, these studies^11^,^12^ did not adjust for confounders. Thirdly, the method of assessment of NAFLD in these studies^11^,^12^ was based on elevated liver enzymes, defined as “above the upper limit of normal”. Liver enzymes do not accurately and reliably predict NALFD and these may be normal in about 80% of such patients.^25^ The two studies^13^,^14^ reporting paradoxical outcomes used either FLI or Controlled attenuation parameter (CAP) on TE. FLI has been shown to have good diagnostic performance to detect NAFLD with a sensitivity and specificity of 81% and 65% respectively.^26^ Similarly, CAP measured on TE has been found to significantly correlate with the degree of steatosis.^25^ Lastly, studies^13^,^14^ with paradoxical outcomes had longer follow-ups and may better represent the effect of NAFLD as compared to the remaining two studies^11^,^12^ which reported only in-hospital outcomes.

The better functional outcomes with NALFD have been attributed to the higher incidence of obesity in NAFLD and the presence of the “obesity paradox”.^27^ Obese patients have long-term low-grade inflammation which provides resistance to inflammatory response as well as hypercatabolic states that is protective against AIS. Better nutritional reserves, higher leptin, and lower adiponectin levels in obese NAFLD patients may also lead to better outcomes. Leptin has shown a neuroprotective effect while adiponectin has been associated with poor functional outcomes after AIS.^13^ It is noteworthy that despite these theoretical possibilities, data on the effect of NAFLD on AIS outcomes is scarce and contradictory. These results should be interpreted with caution till further studies are available. Given that NAFLD and liver fibrosis are part of a single spectrum with the latter being the severe form of the disease, it was not surprising to note worse outcomes after AIS in the presence of advanced liver fibrosis.

All six studies available for analysis used the same FIB-4 index for identifying liver fibrosis. While the gold standard for diagnosis remains liver biopsy, its invasive nature and associated complications have led to the development of several non-invasive markers.^28^ FIB-4 has been considered one of the best markers for detecting advanced fibrosis.^29^ Another plus point was that most studies were prospective and reported outcomes at three months or > one year. All studies consistently reported an increased risk of mortality with liver fibrosis and three of the four studies reported significantly worse functional outcomes with liver fibrosis. The pooled analysis also demonstrated a 2.2 times increased risk of mortality and a 1.89 times increased risk of functional dependence while limited data did not show any effect on AIS recurrence.

Worse outcomes with liver fibrosis can be due to several reasons. Liver fibrosis can increase the risk of cardiac arrhythmias and worsen systemic atherothrombosis which can increase all-cause mortality.^30^,^31^ The stellate cells of the liver involved in liver fibrogenesis also lead to increased levels of pro-inflammatory and procoagulant factors as well as oxidative stress markers.^32^ The degree of liver fibrosis is also associated with a higher prevalence of cardiovascular risk factors like diabetes, hypertension, metabolic syndrome, and dyslipidemia all of which could add to worse outcomes.^33^

### Strength of study:

Strength of the review is that it is the first review on the topic. We conducted separate analysis for the impact of NAFLD and liver fibrosis on AIS outcomes. Our results suggest that liver fibrosis is associated with worse outcomes in AIS. Hence, it is recommended that clinicians should endeavor to identify such cases for appropriate risk stratification and close monitoring of treatment. Further studies should be conducted if targeted interventions for liver fibrosis can improve outcomes of AIS.

### Limitations:

It includes the scarce literature available for analysis. Meta-analysis was not possible for NAFLD studies and fewer than six studies were available for a meta-analysis for liver fibrosis. The studies themselves had inherent bias with many not reporting adjusted data, reporting small sample sizes, and limited follow-up. Variation in the method of assessment of NAFLD was a major limitation as discussed earlier.

## CONCLUSIONS

Scant evidence exists for the effect of NAFLD and liver fibrosis on AIS outcomes. A paradoxical effect of NAFLD on functional outcomes has been noted which needs confirmation by future studies. Liver fibrosis was found to increase the risk of mortality and functional dependence in AIS. Future studies with larger sample sizes, using similar markers for diagnosis of liver disease, and reporting adjusted data with long-term follow-up are needed.

### Authors’ contributions:

**LY:** Conceived and designed the study and prepared the manuscript.

**JH**, **CQ** and **FS:** Collected the data and performed the analysis and did Review.

All authors have read and approved the final manuscript and are responsible for the integrity of the study.
